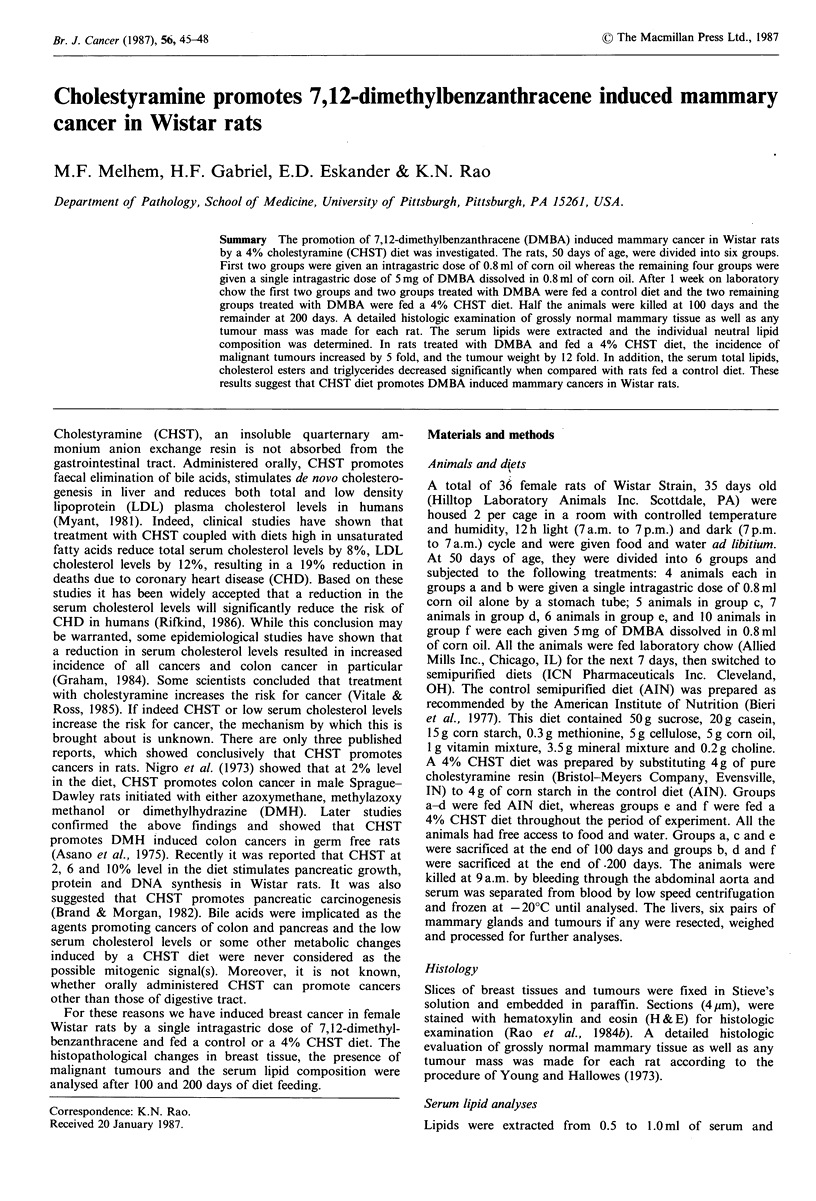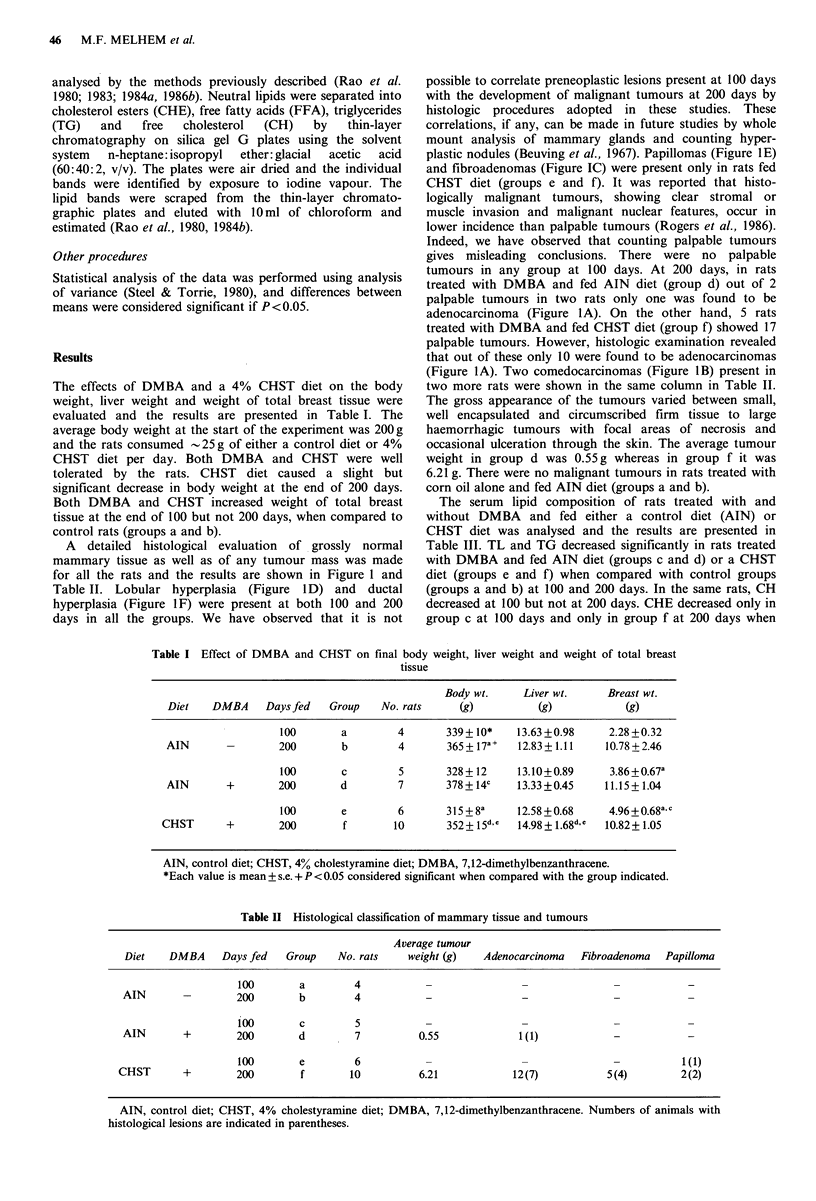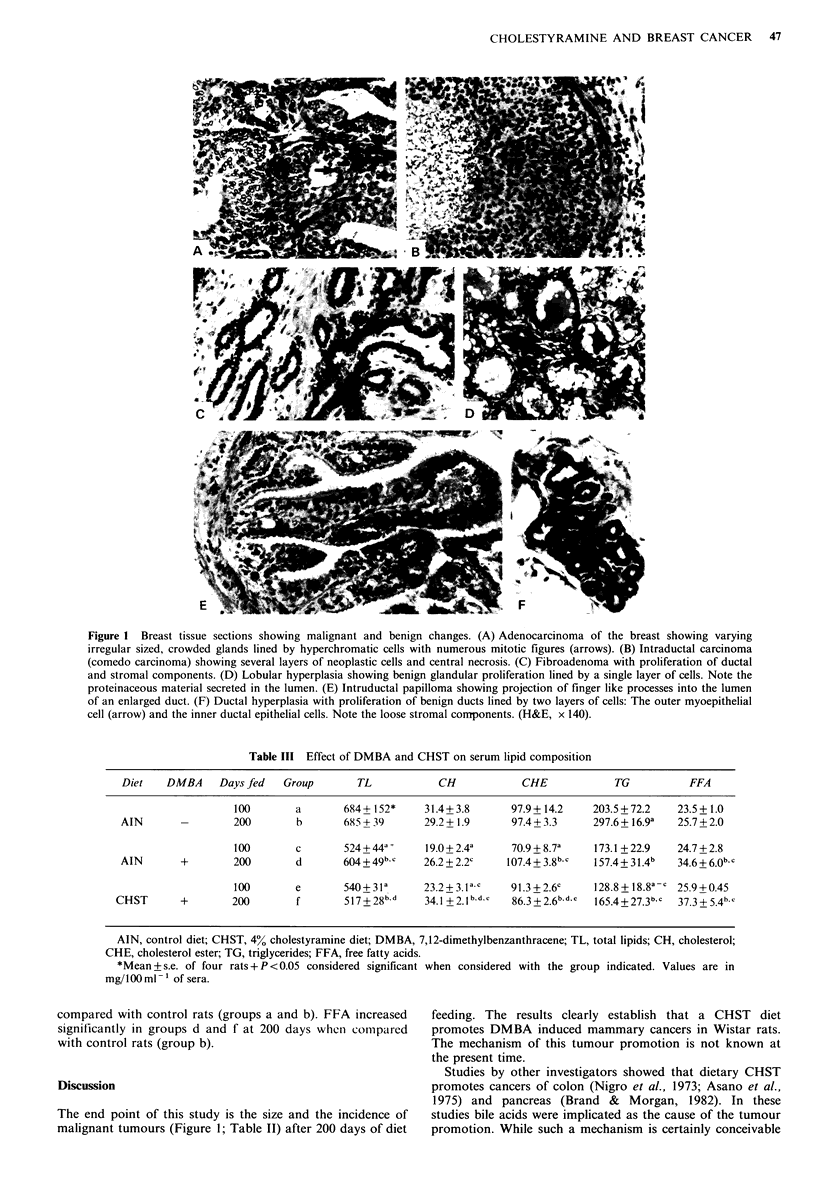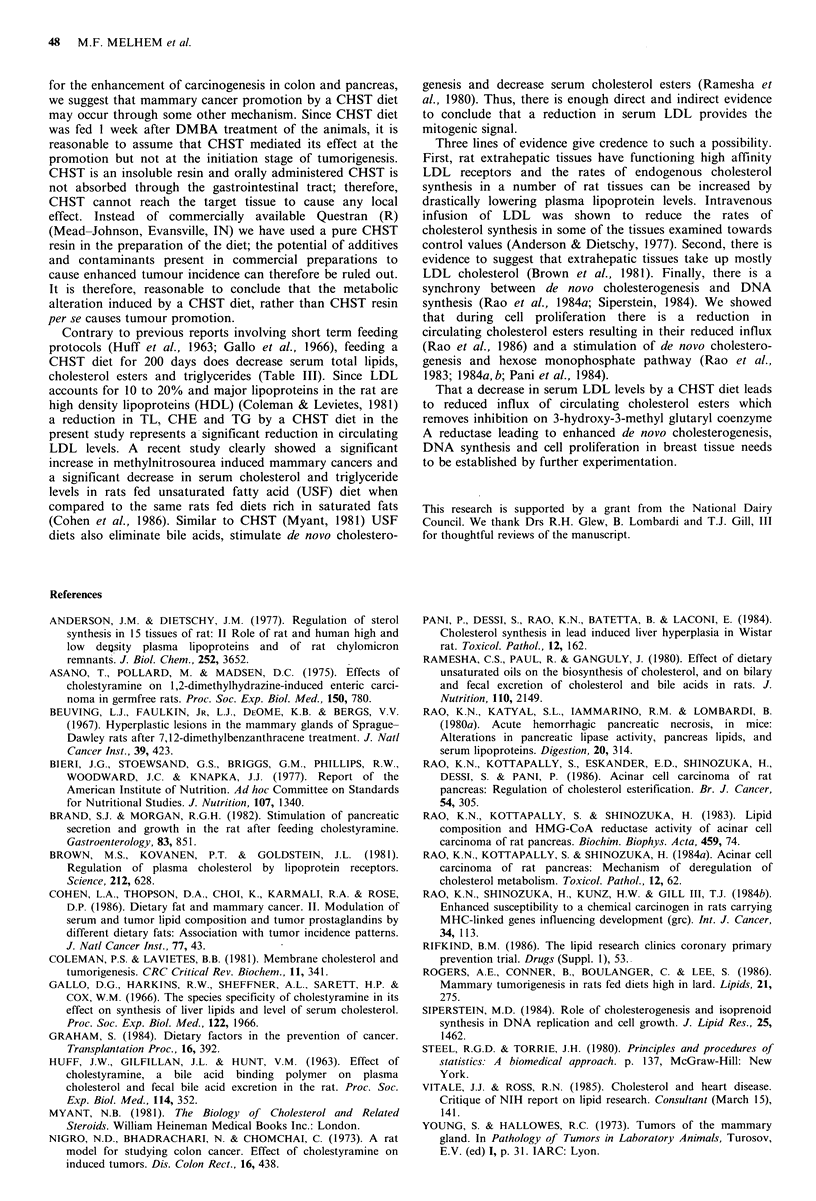# Cholestyramine promotes 7,12-dimethylbenzanthracene induced mammary cancer in Wistar rats.

**DOI:** 10.1038/bjc.1987.150

**Published:** 1987-07

**Authors:** M. F. Melhem, H. F. Gabriel, E. D. Eskander, K. N. Rao

## Abstract

**Images:**


*Br. J. Cancer* (1987), **56**, 45–48
© The Macmillan Press Ltd., 1987

### Cholestyramine promotes 7,12-dimethylbenzanthracene induced mammary cancer in Wistar rats

M.F. Melhem, H.F. Gabriel, E.D. Eskander & K.N. Rao

*Department of Pathology, School of Medicine, University of Pittsburgh, Pittsburgh, PA 15261, USA.*

**Summary** The promotion of 7,12-dimethylbenzanthracene (DMBA) induced mammary cancer in Wistar rats by a 4% cholestyramine (CHST) diet was investigated. The rats, 50 days of age, were divided into six groups. First two groups were given an intragastric dose of 0.8 ml of corn oil whereas the remaining four groups were given a single intragastric dose of 5 mg of DMBA dissolved in 0.8 ml of corn oil. After 1 week on laboratory chow the first two groups and two groups treated with DMBA were fed a control diet and the two remaining groups treated with DMBA were fed a 4% CHST diet. Half the animals were killed at 100 days and the remainder at 200 days. A detailed histologic examination of grossly normal mammary tissue as well as any tumour mass was made for each rat. The serum lipids were extracted and the individual neutral lipid composition was determined. In rats treated with DMBA and fed a 4% CHST diet, the incidence of malignant tumours increased by 5 fold, and the tumour weight by 12 fold. In addition, the serum total lipids, cholesterol esters and triglycerides decreased significantly when compared with rats fed a control diet. These results suggest that CHST diet promotes DMBA induced mammary cancers in Wistar rats.

Cholestyramine (CHST), an insoluble quarternary ammonium anion exchange resin is not absorbed from the gastrointestinal tract. Administered orally, CHST promotes faecal elimination of bile acids, stimulates *de novo* cholesterogenesis in liver and reduces both total and low density lipoprotein (LDL) plasma cholesterol levels in humans (Myant, 1981). Indeed, clinical studies have shown that treatment with CHST coupled with diets high in unsaturated fatty acids reduce total serum cholesterol levels by 8%, LDL cholesterol levels by 12%, resulting in a 19% reduction in deaths due to coronary heart disease (CHD). Based on these studies it has been widely accepted that a reduction in the serum cholesterol levels will significantly reduce the risk of CHD in humans (Rifkind, 1986). While this conclusion may be warranted, some epidemiological studies have shown that a reduction in serum cholesterol levels resulted in increased incidence of all cancers and colon cancer in particular (Graham, 1984). Some scientists concluded that treatment with cholestyramine increases the risk for cancer (Vitale & Ross, 1985). If indeed CHST or low serum cholesterol levels increase the risk for cancer, the mechanism by which this is brought about is unknown. There are only three published reports, which showed conclusively that CHST promotes cancers in rats. Nigro *et al.* (1973) showed that at 2% level in the diet, CHST promotes colon cancer in male Sprague–Dawley rats initiated with either azoxymethane, methylazoxy methanol or dimethylhydrazine (DMH). Later studies confirmed the above findings and showed that CHST promotes DMH induced colon cancers in germ free rats (Asano *et al.*, 1975). Recently it was reported that CHST at 2, 6 and 10% level in the diet stimulates pancreatic growth, protein and DNA synthesis in Wistar rats. It was also suggested that CHST promotes pancreatic carcinogenesis (Brand & Morgan, 1982). Bile acids were implicated as the agents promoting cancers of colon and pancreas and the low serum cholesterol levels or some other metabolic changes induced by a CHST diet were never considered as the possible mitogenic signal(s). Moreover, it is not known, whether orally administered CHST can promote cancers other than those of digestive tract.

For these reasons we have induced breast cancer in female Wistar rats by a single intragastric dose of 7,12-dimethylbenzanthracene and fed a control or a 4% CHST diet. The histopathological changes in breast tissue, the presence of malignant tumours and the serum lipid composition were analysed after 100 and 200 days of diet feeding.

Correspondence: K.N. Rao.

Received 20 January 1987.

### Materials and methods

### *Animals and diets*

A total of 36 female rats of Wistar Strain, 35 days old (Hilltop Laboratory Animals Inc. Scottdale, PA) were housed 2 per cage in a room with controlled temperature and humidity, 12 h light (7 a.m. to 7 p.m.) and dark (7 p.m. to 7 a.m.) cycle and were given food and water *ad libitium*. At 50 days of age, they were divided into 6 groups and subjected to the following treatments: 4 animals each in groups a and b were given a single intragastric dose of 0.8 ml corn oil alone by a stomach tube; 5 animals in group c, 7 animals in group d, 6 animals in group e, and 10 animals in group f were each given 5 mg of DMBA dissolved in 0.8 ml of corn oil. All the animals were fed laboratory chow (Allied Mills Inc., Chicago, IL) for the next 7 days, then switched to semipurified diets (ICN Pharmaceuticals Inc. Cleveland, OH). The control semipurified diet (AIN) was prepared as recommended by the American Institute of Nutrition (Bieri *et al.*, 1977). This diet contained 50 g sucrose, 20 g casein, 15 g corn starch, 0.3 g methionine, 5 g cellulose, 5 g corn oil, 1 g vitamin mixture, 3.5 g mineral mixture and 0.2 g choline. A 4% CHST diet was prepared by substituting 4 g of pure cholestyramine resin (Bristol–Meyers Company, Evensville, IN) to 4 g of corn starch in the control diet (AIN). Groups a–d were fed AIN diet, whereas groups e and f were fed a 4% CHST diet throughout the period of experiment. All the animals had free access to food and water. Groups a, c and e were sacrificed at the end of 100 days and groups b, d and f were sacrificed at the end of 200 days. The animals were killed at 9 a.m. by bleeding through the abdominal aorta and serum was separated from blood by low speed centrifugation and frozen at −20°C until analysed. The livers, six pairs of mammary glands and tumours if any were resected, weighed and processed for further analyses.

### *Histology*

Slices of breast tissues and tumours were fixed in Stieve's solution and embedded in paraffin. Sections (4 $\mu$m), were stained with hematoxylin and eosin (H & E) for histologic examination (Rao *et al.*, 1984*b*). A detailed histologic evaluation of grossly normal mammary tissue as well as any tumour mass was made for each rat according to the procedure of Young and Hallowes (1973).

### *Serum lipid analyses*

Lipids were extracted from 0.5 to 1.0 ml of serum and analysed by the methods previously described (Rao *et al.* 1980; 1983; 1984*a*, 1986*b*). Neutral lipids were separated into cholesterol esters (CHE), free fatty acids (FFA), triglycerides (TG) and free cholesterol (CH) by thin-layer chromatography on silica gel G plates using the solvent system n-heptane:isopropyl ether:glacial acetic acid (60:40:2, v/v). The plates were air dried and the individual bands were identified by exposure to iodine vapour. The lipid bands were scraped from the thin-layer chromatographic plates and eluted with 10 ml of chloroform and estimated (Rao *et al.*, 1980, 1984*b*).

*Other procedures*

Statistical analysis of the data was performed using analysis of variance (Steel & Torrie, 1980), and differences between means were considered significant if $P<0.05$.

### Results

The effects of DMBA and a 4% CHST diet on the body weight, liver weight and weight of total breast tissue were evaluated and the results are presented in Table I. The average body weight at the start of the experiment was 200 g and the rats consumed ~25 g of either a control diet or 4% CHST diet per day. Both DMBA and CHST were well tolerated by the rats. CHST diet caused a slight but significant decrease in body weight at the end of 200 days. Both DMBA and CHST increased weight of total breast tissue at the end of 100 but not 200 days, when compared to control rats (groups a and b).

A detailed histological evaluation of grossly normal mammary tissue as well as of any tumour mass was made for all the rats and the results are shown in Figure 1 and Table II. Lobular hyperplasia (Figure 1D) and ductal hyperplasia (Figure 1F) were present at both 100 and 200 days in all the groups. We have observed that it is not possible to correlate preneoplastic lesions present at 100 days with the development of malignant tumours at 200 days by histologic procedures adopted in these studies. These correlations, if any, can be made in future studies by whole mount analysis of mammary glands and counting hyperplastic nodules (Beuving *et al.*, 1967). Papillomas (Figure 1E) and fibroadenomas (Figure IC) were present only in rats fed CHST diet (groups e and f). It was reported that histologically malignant tumours, showing clear stromal or muscle invasion and malignant nuclear features, occur in lower incidence than palpable tumours (Rogers *et al.*, 1986). Indeed, we have observed that counting palpable tumours gives misleading conclusions. There were no palpable tumours in any group at 100 days. At 200 days, in rats treated with DMBA and fed AIN diet (group d) out of 2 palpable tumours in two rats only one was found to be adenocarcinoma (Figure 1A). On the other hand, 5 rats treated with DMBA and fed CHST diet (group f) showed 17 palpable tumours. However, histologic examination revealed that out of these only 10 were found to be adenocarcinomas (Figure 1A). Two comedocarcinomas (Figure 1B) present in two more rats were shown in the same column in Table II. The gross appearance of the tumours varied between small, well encapsulated and circumscribed firm tissue to large haemorrhagic tumours with focal areas of necrosis and occasional ulceration through the skin. The average tumour weight in group d was 0.55 g whereas in group f it was 6.21 g. There were no malignant tumours in rats treated with corn oil alone and fed AIN diet (groups a and b).

The serum lipid composition of rats treated with and without DMBA and fed either a control diet (AIN) or CHST diet was analysed and the results are presented in Table III. TL and TG decreased significantly in rats treated with DMBA and fed AIN diet (groups c and d) or a CHST diet (groups e and f) when compared with control groups (groups a and b) at 100 and 200 days. In the same rats, CH decreased at 100 but not at 200 days. CHE decreased only in group c at 100 days and only in group f at 200 days when

**Table I** Effect of DMBA and CHST on final body weight, liver weight and weight of total breast tissue

| Diet | DMBA | Days fed | Group | No. rats | Body wt. (g) | Liver wt. (g) | Breast wt. (g) |
|---|---|---|---|---|---|---|---|
| AIN | – | 100 | a | 4 | $339\pm10$* | $13.63\pm0.98$ | $2.28\pm0.32$ |
| | | 200 | b | 4 | $365\pm17^{a+}$ | $12.83\pm1.11$ | $10.78\pm2.46$ |
| AIN | + | 100 | c | 5 | $328\pm12$ | $13.10\pm0.89$ | $3.86\pm0.67^{a}$ |
| | | 200 | d | 7 | $378\pm14^{c}$ | $13.33\pm0.45$ | $11.15\pm1.04$ |
| CHST | + | 100 | e | 6 | $315\pm8^{a}$ | $12.58\pm0.68$ | $4.96\pm0.68^{a,c}$ |
| | | 200 | f | 10 | $352\pm15^{d,e}$ | $14.98\pm1.68^{d,e}$ | $10.82\pm1.05$ |

AIN, control diet; CHST, 4% cholestyramine diet; DMBA, 7,12-dimethylbenzanthracene.
*Each value is mean ± s.e. + $P<0.05$ considered significant when compared with the group indicated.

**Table II** Histological classification of mammary tissue and tumours

| Diet | DMBA | Days fed | Group | No. rats | Average tumour weight (g) | Adenocarcinoma | Fibroadenoma | Papilloma |
|---|---|---|---|---|---|---|---|---|
| AIN | – | 100 | a | 4 | – | – | – | – |
| | | 200 | b | 4 | – | – | – | – |
| AIN | + | 100 | c | 5 | – | – | – | – |
| | | 200 | d | 7 | 0.55 | 1(1) | – | – |
| CHST | + | 100 | e | 6 | – | – | – | 1(1) |
| | | 200 | f | 10 | 6.21 | 12(7) | 5(4) | 2(2) |

AIN, control diet; CHST, 4% cholestyramine diet; DMBA, 7,12-dimethylbenzanthracene. Numbers of animals with histological lesions are indicated in parentheses.

**Figure 1** Breast tissue sections showing malignant and benign changes. (A) Adenocarcinoma of the breast showing varying irregular sized, crowded glands lined by hyperchromatic cells with numerous mitotic figures (arrows). (B) Intraductal carcinoma (comedo carcinoma) showing several layers of neoplastic cells and central necrosis. (C) Fibroadenoma with proliferation of ductal and stromal components. (D) Lobular hyperplasia showing benign glandular proliferation lined by a single layer of cells. Note the proteinaceous material secreted in the lumen. (E) Intruductal papilloma showing projection of finger like processes into the lumen of an enlarged duct. (F) Ductal hyperplasia with proliferation of benign ducts lined by two layers of cells: The outer myoepithelial cell (arrow) and the inner ductal epithelial cells. Note the loose stromal components. (H&E, ×140).

**Table III** Effect of DMBA and CHST on serum lipid composition

| *Diet* | *DMBA* | *Days fed* | *Group* | *TL* | *CH* | *CHE* | *TG* | *FFA* |
|---|---|---|---|---|---|---|---|---|
| AIN | – | 100 | a | $684 \pm 152$* | $31.4 \pm 3.8$ | $97.9 \pm 14.2$ | $203.5 \pm 72.2$ | $23.5 \pm 1.0$ |
| | | 200 | b | $685 \pm 39$ | $29.2 \pm 1.9$ | $97.4 \pm 3.3$ | $297.6 \pm 16.9^{a}$ | $25.7 \pm 2.0$ |
| AIN | + | 100 | c | $524 \pm 44^{a+}$ | $19.0 \pm 2.4^{a}$ | $70.9 \pm 8.7^{a}$ | $173.1 \pm 22.9$ | $24.7 \pm 2.8$ |
| | | 200 | d | $604 \pm 49^{b,c}$ | $26.2 \pm 2.2^{c}$ | $107.4 \pm 3.8^{b,c}$ | $157.4 \pm 31.4^{b}$ | $34.6 \pm 6.0^{b,c}$ |
| CHST | + | 100 | e | $540 \pm 31^{a}$ | $23.2 \pm 3.1^{a,c}$ | $91.3 \pm 2.6^{c}$ | $128.8 \pm 18.8^{a-c}$ | $25.9 \pm 0.45$ |
| | | 200 | f | $517 \pm 28^{b,d}$ | $34.1 \pm 2.1^{b,d,e}$ | $86.3 \pm 2.6^{b,d,e}$ | $165.4 \pm 27.3^{b,c}$ | $37.3 \pm 5.4^{b,c}$ |

AIN, control diet; CHST, 4% cholestyramine diet; DMBA, 7,12-dimethylbenzanthracene; TL, total lipids; CH, cholesterol; CHE, cholesterol ester; TG, triglycerides; FFA, free fatty acids.

*Mean ± s.e. of four rats + $P < 0.05$ considered significant when considered with the group indicated. Values are in mg/100 ml$^{-1}$ of sera.

compared with control rats (groups a and b). FFA increased significantly in groups d and f at 200 days when compared with control rats (group b).

### Discussion

The end point of this study is the size and the incidence of malignant tumours (Figure 1; Table II) after 200 days of diet feeding. The results clearly establish that a CHST diet promotes DMBA induced mammary cancers in Wistar rats. The mechanism of this tumour promotion is not known at the present time.

Studies by other investigators showed that dietary CHST promotes cancers of colon (Nigro *et al.*, 1973; Asano *et al.*, 1975) and pancreas (Brand & Morgan, 1982). In these studies bile acids were implicated as the cause of the tumour promotion. While such a mechanism is certainly conceivable for the enhancement of carcinogenesis in colon and pancreas, we suggest that mammary cancer promotion by a CHST diet may occur through some other mechanism. Since CHST diet was fed 1 week after DMBA treatment of the animals, it is reasonable to assume that CHST mediated its effect at the promotion but not at the initiation stage of tumorigenesis. CHST is an insoluble resin and orally administered CHST is not absorbed through the gastrointestinal tract; therefore, CHST cannot reach the target tissue to cause any local effect. Instead of commercially available Questran (R) (Mead–Johnson, Evansville, IN) we have used a pure CHST resin in the preparation of the diet; the potential of additives and contaminants present in commercial preparations to cause enhanced tumour incidence can therefore be ruled out. It is therefore, reasonable to conclude that the metabolic alteration induced by a CHST diet, rather than CHST resin *per se* causes tumour promotion.

Contrary to previous reports involving short term feeding protocols (Huff *et al.*, 1963; Gallo *et al.*, 1966), feeding a CHST diet for 200 days does decrease serum total lipids, cholesterol esters and triglycerides (Table III). Since LDL accounts for 10 to 20% and major lipoproteins in the rat are high density lipoproteins (HDL) (Coleman & Levietes, 1981) a reduction in TL, CHE and TG by a CHST diet in the present study represents a significant reduction in circulating LDL levels. A recent study clearly showed a significant increase in methylnitrosourea induced mammary cancers and a significant decrease in serum cholesterol and triglyceride levels in rats fed unsaturated fatty acid (USF) diet when compared to the same rats fed diets rich in saturated fats (Cohen *et al.*, 1986). Similar to CHST (Myant, 1981) USF diets also eliminate bile acids, stimulate *de novo* cholesterogenesis and decrease serum cholesterol esters (Ramesha *et al.*, 1980). Thus, there is enough direct and indirect evidence to conclude that a reduction in serum LDL provides the mitogenic signal.

Three lines of evidence give credence to such a possibility. First, rat extrahepatic tissues have functioning high affinity LDL receptors and the rates of endogenous cholesterol synthesis in a number of rat tissues can be increased by drastically lowering plasma lipoprotein levels. Intravenous infusion of LDL was shown to reduce the rates of cholesterol synthesis in some of the tissues examined towards control values (Anderson & Dietschy, 1977). Second, there is evidence to suggest that extrahepatic tissues take up mostly LDL cholesterol (Brown *et al.*, 1981). Finally, there is a synchrony between *de novo* cholesterogenesis and DNA synthesis (Rao *et al.*, 1984*a*; Siperstein, 1984). We showed that during cell proliferation there is a reduction in circulating cholesterol esters resulting in their reduced influx (Rao *et al.*, 1986) and a stimulation of *de novo* cholesterogenesis and hexose monophosphate pathway (Rao *et al.*, 1983; 1984*a,b*; Pani *et al.*, 1984).

That a decrease in serum LDL levels by a CHST diet leads to reduced influx of circulating cholesterol esters which removes inhibition on 3-hydroxy-3-methyl glutaryl coenzyme A reductase leading to enhanced *de novo* cholesterogenesis, DNA synthesis and cell proliferation in breast tissue needs to be established by further experimentation.

This research is supported by a grant from the National Dairy Council. We thank Drs R.H. Glew, B. Lombardi and T.J. Gill, III for thoughtful reviews of the manuscript.

### References

ANDERSON, J.M. & DIETSCHY, J.M. (1977). Regulation of sterol synthesis in 15 tissues of rat: II Role of rat and human high and low density plasma lipoproteins and of rat chylomicron remnants. *J. Biol. Chem.*, **252**, 3652.

ASANO, T., POLLARD, M. & MADSEN, D.C. (1975). Effects of cholestyramine on 1,2-dimethylhydrazine-induced enteric carcinoma in germfree rats. *Proc. Soc. Exp. Biol. Med.*, **150**, 780.

BEUVING, L.J., FAULKIN, JR, L.J., DEOME, K.B. & BERGS, V.V. (1967). Hyperplastic lesions in the mammary glands of Sprague–Dawley rats after 7,12-dimethylbenzanthracene treatment. *J. Natl Cancer Inst.*, **39**, 423.

BIERI, J.G., STOEWSAND, G.S., BRIGGS, G.M., PHILLIPS, R.W., WOODWARD, J.C. & KNAPKA, J.J. (1977). Report of the American Institute of Nutrition. *Ad hoc* Committee on Standards for Nutritional Studies. *J. Nutrition*, **107**, 1340.

BRAND, S.J. & MORGAN, R.G.H. (1982). Stimulation of pancreatic secretion and growth in the rat after feeding cholestyramine. *Gastroenterology*, **83**, 851.

BROWN, M.S., KOVANEN, P.T. & GOLDSTEIN, J.L. (1981). Regulation of plasma cholesterol by lipoprotein receptors. *Science*, **212**, 628.

COHEN, L.A., THOPSON, D.A., CHOI, K., KARMALI, R.A. & ROSE, D.P. (1986). Dietary fat and mammary cancer. II. Modulation of serum and tumor lipid composition and tumor prostaglandins by different dietary fats: Association with tumor incidence patterns. *J. Natl Cancer Inst.*, **77**, 43.

COLEMAN, P.S. & LAVIETES, B.B. (1981). Membrane cholesterol and tumorigenesis. *CRC Critical Rev. Biochem.*, **11**, 341.

GALLO, D.G., HARKINS, R.W., SHEFFNER, A.L., SARETT, H.P. & COX, W.M. (1966). The species specificity of cholestyramine in its effect on synthesis of liver lipids and level of serum cholesterol. *Proc. Soc. Exp. Biol. Med.*, **122**, 1966.

GRAHAM, S. (1984). Dietary factors in the prevention of cancer. *Transplantation Proc.*, **16**, 392.

HUFF, J.W., GILFILLAN, J.L. & HUNT, V.M. (1963). Effect of cholestyramine, a bile acid binding polymer on plasma cholesterol and fecal bile acid excretion in the rat. *Proc. Soc. Exp. Biol. Med.*, **114**, 352.

MYANT, N.B. (1981). *The Biology of Cholesterol and Related Steroids*. William Heineman Medical Books Inc.: London.

NIGRO, N.D., BHADRACHARI, N. & CHOMCHAI, C. (1973). A rat model for studying colon cancer. Effect of cholestyramine on induced tumors. *Dis. Colon Rect.*, **16**, 438.

PANI, P., DESSI, S., RAO, K.N., BATETTA, B. & LACONI, E. (1984). Cholesterol synthesis in lead induced liver hyperplasia in Wistar rat. *Toxicol. Pathol.*, **12**, 162.

RAMESHA, C.S., PAUL, R. & GANGULY, J. (1980). Effect of dietary unsaturated oils on the biosynthesis of cholesterol, and on bilary and fecal excretion of cholesterol and bile acids in rats. *J. Nutrition*, **110**, 2149.

RAO, K.N., KATYAL, S.L., IAMMARINO, R.M. & LOMBARDI, B. (1980*a*). Acute hemorrhagic pancreatic necrosis, in mice: Alterations in pancreatic lipase activity, pancreas lipids, and serum lipoproteins. *Digestion*, **20**, 314.

RAO, K.N., KOTTAPALLY, S., ESKANDER, E.D., SHINOZUKA, H., DESSI, S. & PANI, P. (1986). Acinar cell carcinoma of rat pancreas: Regulation of cholesterol esterification. *Br. J. Cancer*, **54**, 305.

RAO, K.N., KOTTAPALLY, S. & SHINOZUKA, H. (1983). Lipid composition and HMG-CoA reductase activity of acinar cell carcinoma of rat pancreas. *Biochim. Biophys. Acta*, **459**, 74.

RAO, K.N., KOTTAPALLY, S. & SHINOZUKA, H. (1984*a*). Acinar cell carcinoma of rat pancreas: Mechanism of deregulation of cholesterol metabolism. *Toxicol. Pathol.*, **12**, 62.

RAO, K.N., SHINOZUKA, H., KUNZ, H.W. & GILL III, T.J. (1984*b*). Enhanced susceptibility to a chemical carcinogen in rats carrying MHC-linked genes influencing development (grc). *Int. J. Cancer*, **34**, 113.

RIFKIND, B.M. (1986). The lipid research clinics coronary primary prevention trial. *Drugs* (Suppl. 1), 53.

ROGERS, A.E., CONNER, B., BOULANGER, C. & LEE, S. (1986). Mammary tumorigenesis in rats fed diets high in lard. *Lipids*, **21**, 275.

SIPERSTEIN, M.D. (1984). Role of cholesterogenesis and isoprenoid synthesis in DNA replication and cell growth. *J. Lipid Res.*, **25**, 1462.

STEEL, R.G.D. & TORRIE, J.H. (1980). *Principles and procedures of statistics: A biomedical approach*. p. 137, McGraw-Hill: New York.

VITALE, J.J. & ROSS, R.N. (1985). Cholesterol and heart disease. Critique of NIH report on lipid research. *Consultant* (March 15), 141.

YOUNG, S. & HALLOWES, R.C. (1973). Tumors of the mammary gland. In *Pathology of Tumors in Laboratory Animals*, Turosov, E.V. (ed) **I**, p. 31. IARC: Lyon.